# Genome-Wide Association Study Reveals Novel Loci and Candidate Genes of Vitamin E Content in Sesame (*Sesamum indicum* L.)

**DOI:** 10.3390/antiox15091088

**Published:** 2026-08-29

**Authors:** Zishu Luo, Jianglong Zhou, Yijia Zhang, Huan Li, Rong Zhou, Ting Zhou, Yanxin Zhang, Jun You, Linhai Wang

**Affiliations:** Key Laboratory of Biology and Genetic Improvement of Oil Crops, Ministry of Agriculture and Rural Affairs, Oil Crops Research Institute, Chinese Academy of Agricultural Sciences, Wuhan 430062, China; luozishu22@163.com (Z.L.); zjl274169@163.com (J.Z.); zyj6660221@163.com (Y.Z.); lihuan01@caas.cn (H.L.); zhourong@caas.cn (R.Z.); zhoutingyls@caas.cn (T.Z.); zhangyanxin@caas.cn (Y.Z.)

**Keywords:** sesame, vitamin E, genome-wide association study, transcriptome

## Abstract

Sesame is a significant oilseed crop whose exceptional oxidative stability is closely associated with its abundant endogenous antioxidants. As one of the most predominant lipid-soluble antioxidants in sesame, vitamin E (VE) plays a critical role in scavenging lipid peroxyl radicals, terminating lipid peroxidation chain reactions, and protecting cellular membranes from oxidative damage, thereby maintaining intracellular redox homeostasis and attenuating the development of oxidative stress-related disorders. Although VE is a potent natural antioxidant with significant pharmacological activities in mitigating oxidative stress-related disorders, the genetic mechanisms underlying the natural variation in VE content in sesame remain incompletely understood. Here, variation in VE content was evaluated across 400 sesame accessions grown in two environments. Ultra-high-performance liquid chromatography (UHPLC) analysis revealed that only γ-tocopherol was detected in sesame seeds, with concentrations between 169.33 and 463.31 mg/kg, averaging 316.28 mg/kg. The newly acquired SNP and InDel data from whole-genome resequencing were associated with the phenotypic data, leading to the identification of five significant loci associated with VE content. Comparative transcriptomic profiling of two sesame accessions with distinct VE contents revealed the differential expression of key biosynthetic enzyme genes (such as *GGDR*, *PDS1*, *VTE2*, and *VTE4*) between the two accessions. By integrating a genome-wide association study with transcriptomic data, two primary candidate effector genes, *SINPZ1100015*/*SiNST1* and *SINPZ0901927*, were identified, with *SiNST1* being particularly prominent. Functional validation further showed that overexpression of *SiNST1* in the hairy root of sesame significantly decreased VE content. This investigation advances the comprehension of variations in VE content and the regulatory mechanisms governing its biosynthetic metabolism in sesame, supporting the development of functional sesame varieties for dietary antioxidant supplementation.

## 1. Introduction

Sesame (*Sesamum indicum* L.), an essential oilseed crop in the *Sesamaceae* family, is predominantly cultivated in tropical and subtropical regions of Asia and Africa [1,2]. Sesame is widely utilized for oil extraction or direct consumption, and its strong antioxidant properties also render it broadly applicable in pharmaceuticals and chemicals [3]. Sesame seeds are particularly rich in vitamin E (VE), with black sesame seeds containing VE content as high as 50.4 mg/100 g [4]. VE is an essential fat-soluble vitamin that cannot be synthesized autonomously in humans and animals. As a potent natural antioxidant, it effectively mitigates ROS-induced oxidative damage to cells and lipids [5], thereby inhibiting lipid peroxidation and preserving biological membrane integrity. The imbalance between ROS production and antioxidant defense systems leads to oxidative stress, a common pathological basis underlying various human chronic diseases [6,7]. VE is thought to act as an antioxidant and anti-inflammatory and is involved in numerous aspects of human health, including anticancer, anti-atherogenic, cardioprotective and neuroprotective activities [8,9,10,11]. Given its crucial role in mitigating oxidative damage, enhancing VE content in dietary sources represents a promising strategy for improving human health through nutritional intervention. Although sesame is recognized as a crop rich in VE, the underlying mechanisms that govern VE biosynthesis in sesame remain poorly understood. This knowledge gap has hindered efforts to develop new sesame varieties with enhanced antioxidant potential and medicinal value.

First identified in 1922, VE is also known as tocopherol, a designation originating from its recognized role in fertility, with deficiency causing reproductive failure in animals and humans [12]. The VE family comprises eight forms, including α, β, γ, δ-tocopherols and α, β, γ, δ-tocotrienols, distinguished primarily by the saturation of their hydrophobic tails and the methyl groups’ arrangement on the aromatic rings [13]. Sesame seeds predominantly contain γ-tocopherol, comprising over 95% of their total tocopherol content [14]. The biosynthetic pathway of VE in higher plants has been gradually improved with the cloning and functional validation of key enzyme genes for VE biosynthesis in the model plant *Arabidopsis thaliana* [15]. The primary precursors of VE include homogentisic acid (HGA), phytyl diphosphate (PDP), and geranylgeranyl diphosphate (GGDP), derived from the shikimate acid pathway, chlorophyll degradation, and the methylerythritol phosphate pathway, respectively [13,16]. The core route begins with the enzymatic reduction of p-hydroxyphenylpyruvate (HPP) to HGA by 4-HPP dioxygenase (HPPD), followed by decarboxylation and condensation of HGA with PDP via homogentisate phytyltransferase (HPT) to produce 2-methyl-6-phytyl-1,4-benzoquinol (MPBQ). From MPBQ, the pathway diverges: MPBQ is methylated to 2,3-dimethyl-6-phytyl-1,4-benzoquinol (DMPBQ) by 2-methyl-6-phytyl-1,4-benzoquinol methyltransferase (MPBQ MT) and then cyclized by tocopherol cyclase (TC) to form γ-tocopherol, which is further methylated by γ-tocopherol methyltransferase (γ-TMT) to produce α-tocopherol; alternatively, MPBQ is directly cyclized by TC to yield δ-tocopherol, which is subsequently converted to β-tocopherol [11,13,14].

As sequencing costs decline, genome-wide association studies (GWAS) are increasingly used to analyze the genetic basis of complex agronomic traits and identify superior allelic variations. This has greatly promoted basic research and the molecular breeding of crops. GWAS has been successful in identifying significant associations for VE in many crops, including oil palm [17], sweet corn [18,19], tomato [20], maize [21,22], rapeseed [23], rice [24] and soybean [25]. Among these, Kazemzadeh et al. [24] conducted a GWAS on 179 genotypically diverse rice germplasms with 34,323 single nucleotide polymorphism (SNP) markers and identified a total of 13 quantitative trait loci (QTLs) associated with the contents of total tocopherols and α-tocopherol in rice grains. Li et al. [19] performed a comprehensive GWAS on 295 inbred sweet corn lines, identifying 13 QTLs associated with tocopherol-related traits and elucidating the roles of three genes—*ZmCS2*, *Zmshki1* and *ZmB4FMV1*—in regulating α-tocopherol accumulation in kernels. Wang et al. [23] detected a QTL, *qVE.C02*, associated with total tocopherols and α-tocopherol contents in *Brassica napus* (oilseed rape), which exhibited a phenotypic variance explanation of up to 18.71%. In sesame, He et al. [26] conducted a GWAS with 5962 genome-wide loci from 96 sesame accessions, identifying LG08_6621957 on chromosome 8 as significantly associated with γ-tocopherol. The limited sample size and scarcity of high-density polymorphic loci in current research hinder comprehensive and precise genetic profiling of the VE variation in sesame seeds. Consequently, the genetic architecture underlying the natural variation in this crucial antioxidant component in sesame remains largely unexplored.

In the present study, 400 sesame accessions cultivated under two distinct environmental conditions were investigated, and GWAS was performed to identify significant loci and candidate genes regulating VE content in sesame. Transcriptomic profiling of two sesame accessions with contrasting VE levels, integrated with GWAS results, revealed that *SiNST1* is a key regulatory gene involved in VE biosynthesis. Unlike previous studies with limited sample sizes, this study combines a large association panel, multi-environment phenotyping, and transcriptomic profiling to dissect the genetic architecture of VE content in sesame. Collectively, these integrated analyses aim to identify key genetic regulators of VE biosynthesis that can inform future strategies for enhancing seed antioxidant capacity in sesame, thereby contributing to the broader objective of developing functional varieties for dietary antioxidant supplementation.

## 2. Materials and Methods

### 2.1. Plant Materials

A collection of 400 sesame accessions, including 318 landraces and 82 cultivars (Table S1), was sourced from the National Mid-term Gene Bank for Oil Crops at the Oil Crops Research Institute, Chinese Academy of Agricultural Sciences (OCRI-CAAS), Wuhan, China, and evaluated in field trials at two locations (Wuhan, Hubei Province and Linqing, Shandong Province). Wuhan site is located in central China, in the middle reaches of the Yangtze River, and has a subtropical humid monsoon climate. The previous crop of Wuhan site was rapeseed. Linqing site is located in the central part of the North China Plain and has a semi-humid continental climate. The previous crop of Linqing site was maize. At each location, a randomized block design with three replications was employed, with each replication consisting of 30 plants per accession in a 2 m × 1 m plot. For VE content determination, the three replicates of each accession in the same location were mixed in equal amounts, and three mixed samples of each accession from each location were measured.

### 2.2. Determination of Vitamin E (VE) Content in Sesame Seed

VE content in sesame seeds was analyzed using normal-phase ultra-high-performance liquid chromatography (UHPLC; Waters Corporation, Milford, MA, USA) with an external standard method according to previous reports with minor modifications, and all values are expressed as mg/kg dry seed weight. Briefly, approximately 0.03–0.05 g of sesame seeds was placed in a centrifuge tube with two 5 mm ferrite beads and 1.5 mL of n-hexane (Sinopharm Chemical Reagent Co., Ltd., Shanghai, China). The mixture was shaken for 5 min to ensure thorough contact between the n-hexane and sesame seeds. To prevent volatilization, the sample was sealed with tin foil and cling film and then shaken for 2–3 h, followed by extraction for 16–24 h. The extract underwent centrifugation at 12,000 rpm for 10 min (Thermo Fisher Scientific, Waltham, MA, USA) and was filtered using a 0.22 μm organic-phase filter membrane (SCAA-104, ANPEL, Shanghai, China). The supernatant was then collected for analysis [27,28].

The UHPLC was performed using an Agilent ZORBAX RX-SIL column (4.6 × 250 mm, 5-Micron; Agilent Technologies, Santa Clara, CA, USA) with a mobile phase of n-hexane and isopropanol (99:1). The flow rate was established at 1 mL/min, with an injection volume set at 5 µL. The column temperature was held at 30 °C, and detection occurred at a wavelength of 292 nm.

### 2.3. GWAS and Haplotype Analysis

The GWAS analysis utilized 1,383,209 SNPs and 249,075 InDels, each with a minor allele frequency (MAF) of at least 0.05 [29]. Phenotypic and genotypic association analyses utilized three models: Mixed Linear Model (MLM), Fixed and Random Model Circulating Probability Unification (FarmCPU), and the 3 Variance-component Multi-locus Random-SNP-effect Mixed Linear Model (3VmrMLM). The top five principal components (PCs) were used as covariates in all models to control for population structure. MLM and FarmCPU analyses were performed using the rMVP software package v1.4.6 [30], setting −log10(*P*) ≥ 5 as the threshold based on the effective number of independent markers (n = 149,769) calculated using GEC software v0.2 [31]. The 3VmrMLM analysis utilized the IIIVmrMLM package v1.0 with the Multi_env method [32], applying a LOD threshold of ≥3 to identify significant SNPs and InDels. The proportion of phenotypic variance explained by a given SNP was estimated as described by Shim et al. [33]. Candidate genes were identified within the ±89 kb linkage disequilibrium (LD) window of each peak locus [29].

### 2.4. Transcriptome Analysis

For transcriptome analysis, one high-VE-content (HE) and one low-VE-content (LE) sesame accession were selected. Seed samples were collected at 10, 20, and 30 days post-anthesis (DPA), rapidly frozen in liquid nitrogen and stored at −80 °C. Total RNA extraction was performed utilizing the EASYspin Plus Plant RNAKit (Vazyme Biotech, Nanjing, China). The RNA-seq libraries were prepared by Novogene Corporation (Beijing, China), and paired-end sequencing was performed on the DNBSEQ-T7 platform. Raw paired-end reads were trimmed and quality-controlled by fastp v1.3.5 with default parameters [34]. Clean reads were mapped to the sesame “Zhongzhi 13” reference genome [35] using HISAT2 v2.2.3 [36], and gene-level read counts were obtained with featureCounts v2.1.1 [37] and normalized as transcripts per million (TPM). Differentially expressed genes (DEGs) were identified using DESeq2 v1.50.2 [38] with an adjusted *P*-value threshold of 0.05 and an absolute fold change threshold of 2. Statistical analyses and mapping results of RNA-seq reads are presented in Table S2.

### 2.5. Identification and Expression Analysis of Genes Involved in the VE Biosynthesis Pathway in Sesame

The genes implicated in VE biosynthesis in *Arabidopsis* were sourced from the study by Niu et al. [15]. Homologous genes in sesame were identified by BLASTP searches against the sesame reference genome using an E-value cutoff of 1 × 10^−5^, minimum identity ≥ 30%, and query coverage ≥ 50%. The expression levels of the identified sesame homologs were extracted from the transcriptome data generated in this study. To visualize the expression patterns of VE pathway genes across the three developmental stages (10, 20, and 30 DPA) in both the HE and LE accessions, a heatmap was generated in R v3.5.0 using the pheatmap package based on log2-transformed TPM values.

### 2.6. qRT-PCR

qRT-PCR was carried out on three biological replicates using the ChamQ™ SYBR1 qPCR Master Mix (Vazyme Biotech, Nanjing, China) on a LightCycler480 (Roche Diagnostics International Ltd., Rotkreuz, Switzerland) real-time PCR system. Specific primers for the target genes were designed with the help of Primer Premier 5.0 (PREMIER Biosoft, San Francisco, CA, USA). The relative expression levels of each gene were assessed utilizing the 2^−ΔΔCT^ method [39]. The sesame *Histone3.3* gene (*SINPZ1301428*) served as an internal control [40]. The gene-specific primers used in this study are listed in Table S3.

### 2.7. Functional Verification of SiNST1 in Sesame Hairy Roots

Coding sequences for *SiNST1* (harboring C allele at locus SNP11:142842) were obtained from the sesame variety Zhongzhi13 using RT-PCR with gene-specific primers provided in Table S3. The PCR product was subsequently cloned into the pKR203S vector, regulated by the constitutive 35S promoter. The pKR203S vector contains independently expressed superfolder GFP (sfGFP) as a selection marker. Subsequently, the sequencing-verified recombinant plasmid pKR203S-*SiNST1* was transformed into *Agrobacterium rhizogenes* K599 (Shanghai Weidi Biotechnology Co., Ltd., Shanghai, China). Sesame seeds were cultured in MS medium, and cotyledons from seedlings grown for 7–10 days were subjected to Agrobacterium-mediated infection according to the methods previously described [41]. Positive hairy root lines were selected via fluorescence screening, and qRT-PCR analysis further confirmed the expression levels of *SiNST1*. Hairy roots were freeze-dried prior to analysis (MM 400, Retsch, Haan, Germany), and the VE content in *SiNST1*-overexpressing root lines was determined by the UHPLC method described above.

### 2.8. Statistical Analysis

Statistical analysis of the phenotypic data involved two-tailed unpaired Student’s *t*-tests and one-way ANOVA with the least significant difference (LSD) multiple range test, performed using IBM SPSS 26.0. Correlation analysis and frequency distribution were conducted using R.

## 3. Results

### 3.1. Statistical and Variability Analysis of VE Content of Sesame Seeds

**Figure 1:**
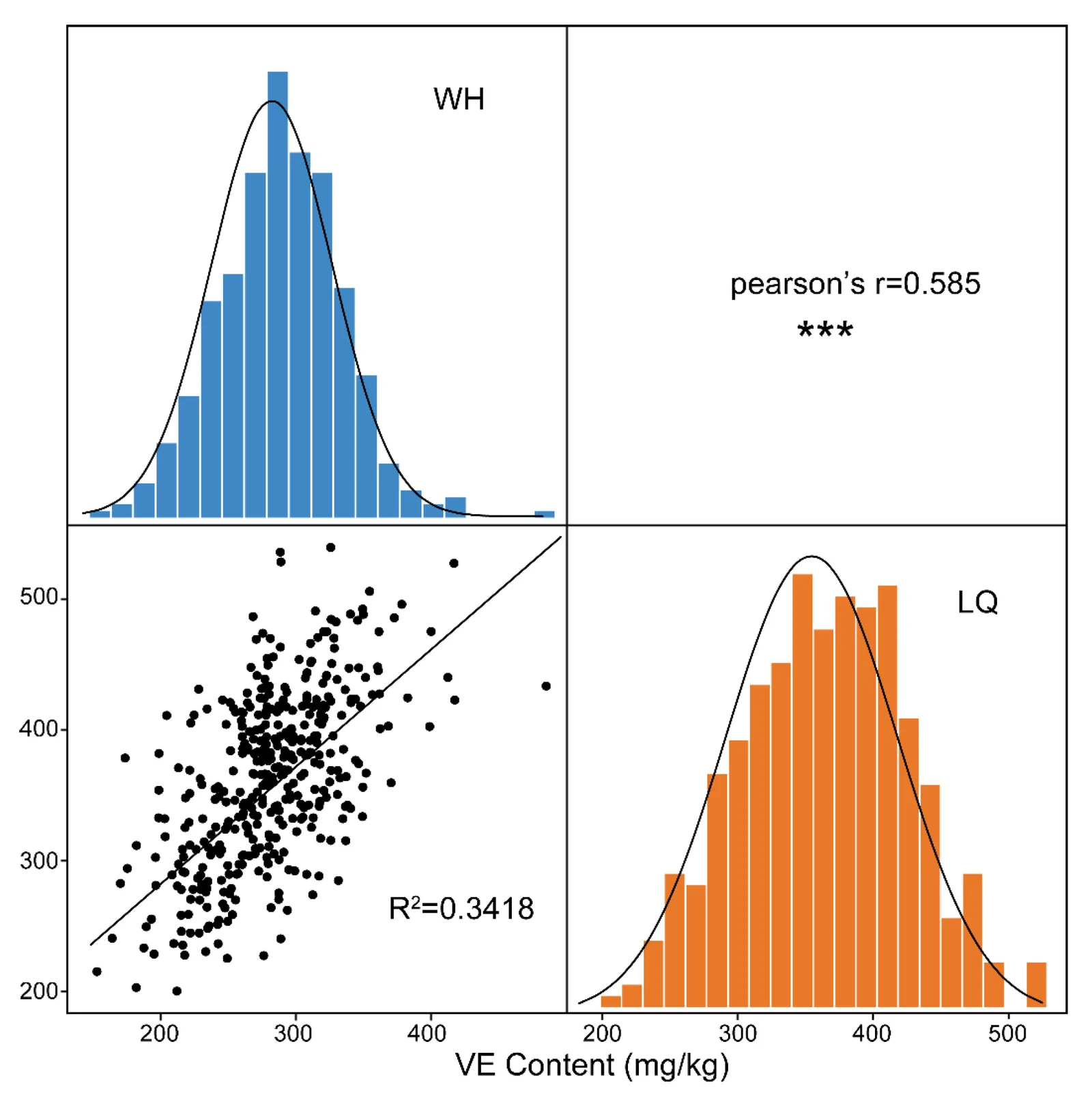
Variation and correlation of VE content in sesame natural population under different environments. WH, Wuhan; LQ, Linqing. Black dots and colored bars represent the dispersion and frequency distribution of the phenotypic data. ***, *P* < 0.001.

To investigate the natural variation in VE content among sesame germplasm resources, the VE content in the seeds of 400 sesame accessions from two environmental conditions (Wuhan and Linqing) was analyzed (Table S4). The findings revealed significant phenotypic variation in VE content within the natural population, displaying a normal distribution. This suggests that VE content in sesame is a quantitative trait influenced by multiple genes. Specifically, in the Wuhan environment, the VE content of the sesame population ranged from 151.59 to 483.26 mg/kg, with a mean value of 279.17 ± 45.67 mg/kg and a coefficient of variation (CV) of 16.36%. In contrast, the sesame population grown in Linqing exhibited generally higher VE content, with values spanning from 199.52 to 523.00 mg/kg, a mean of 354.62 ± 62.87 mg/kg, and a CV of 17.73% (Table 1). When calculating the average VE content across the two environments, the 400 sesame accessions showed a range of 169.33 to 463.31 mg/kg and an overall mean of 316.28 ± 48.83 mg/kg. These findings provide a valuable reference for the subsequent screening and selection of extreme germplasm accessions with high or low VE content. Analysis showed a significant positive correlation between VE content in the two environments, with a correlation coefficient of 0.58 (*P* < 0.001). This significant positive correlation suggests reasonable cross-environmental consistency of VE content, supporting the reliability of the phenotypic data in this study (Figure 1).

### 3.2. GWAS Identifies Significant SNPs and InDels Associated with VE Content

**Figure 2:**
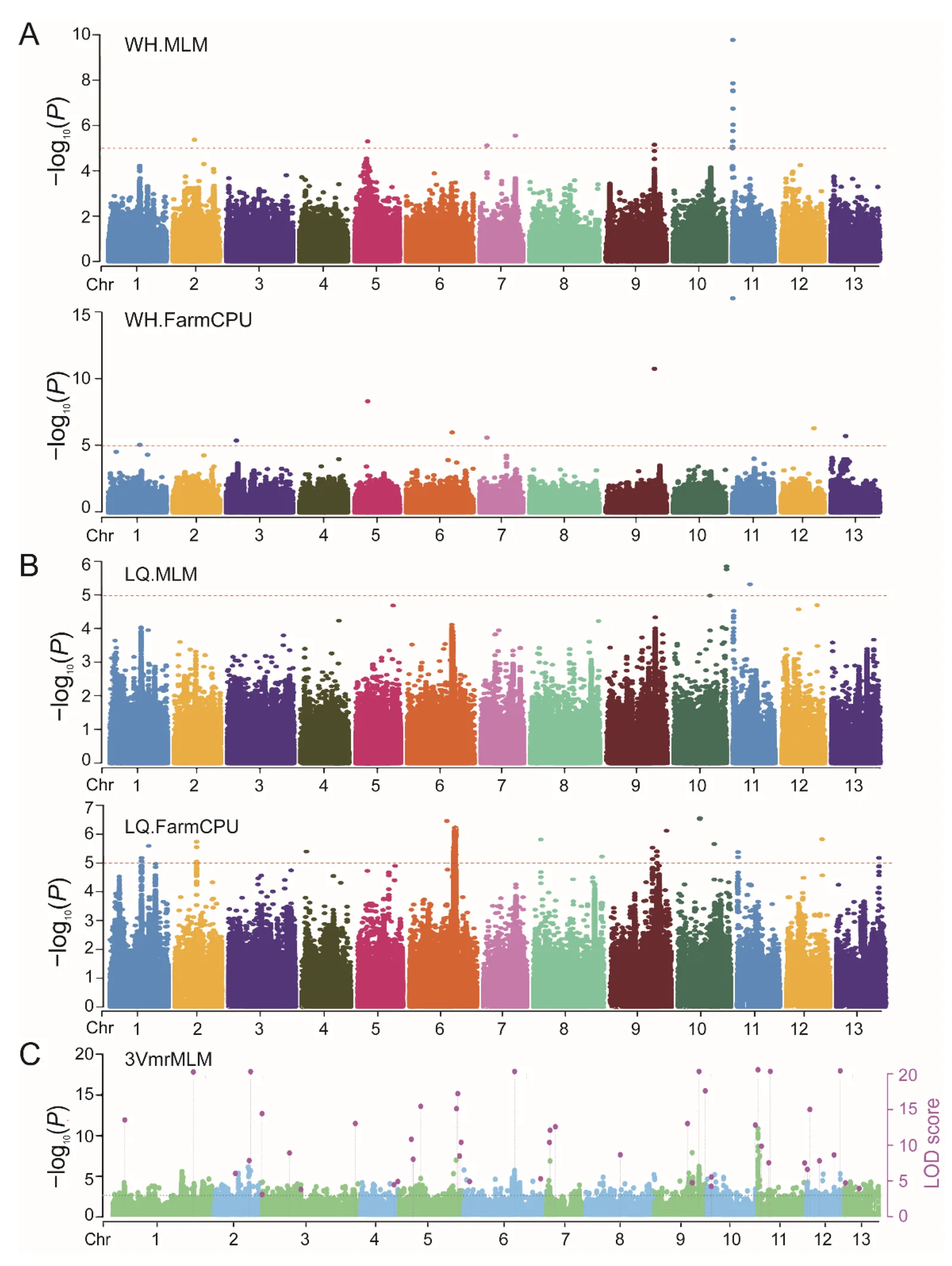
Manhattan plots showing marker-trait associations for VE content. (**A**) GWAS results for VE in Wuhan environment (WH). (**B**) GWAS results for VE in Linqing environment (LQ). (**C**) GWAS results for VE by 3VmrMLM. The red dashed line indicates the threshold −log10(*P*) = 5 for MLM and FarmCPU; the black dashed line indicates the threshold or LOD = 3 for 3VmrMLM.

VE phenotypic data collected from two environments were used for GWAS with more than 1.3 million loci using three statistical models (MLM, FarmCPU, and 3VmrMLM). A total of 71 loci significantly associated with VE content (*P* < 10^−5^ or LOD ≥ 3) were identified in two environments. Manhattan and QQ plots of GWAS are shown in Figure 2 and Figure S1, respectively. Specifically, a total of 12 significant loci associated with VE were detected by MLM, including 9 SNPs and 3 InDels; these loci were primarily distributed on chromosomes 2, 5, 7, 9, 10, and 11. In the FarmCPU, 25 significant VE-associated loci (22 SNPs and 3 InDels) were identified, which were spread across all 13 sesame chromosomes. Notably, one locus (SNP11:142842) was detected in both Wuhan and Linqing environments and was thus counted only once in the total set. Among the three models, 3VmrMLM detected the highest number of significant loci, with 45 loci in total, and these loci were also distributed across all 13 chromosomes of sesame.

In Wuhan environment, 10 significant loci associated with VE content were detected by the MLM, while 9 loci were detected by the FarmCPU (Figure 2A). Notably, the most significant locus (with the lowest *P*-value) in both models was SNP11:142842, with corresponding −log10(*P*) values of 9.77 (MLM) and 16.08 (FarmCPU). In contrast, only 2 significant VE-associated loci were detected by the MLM in the Linqing environment, whereas the FarmCPU detected 17 (Figure 2B); the most significant locus in both models under Linqing was SNP10:14960458, with −log10(*P*) values of 5.87 (MLM) and 6.55 (FarmCPU), respectively. For the 3VmrMLM, a total of 45 significant loci were detected across multiple environments (Figure 2C), among which SNP11:142842 exhibited the strongest association (LOD = 52.09).

**Figure 3:**
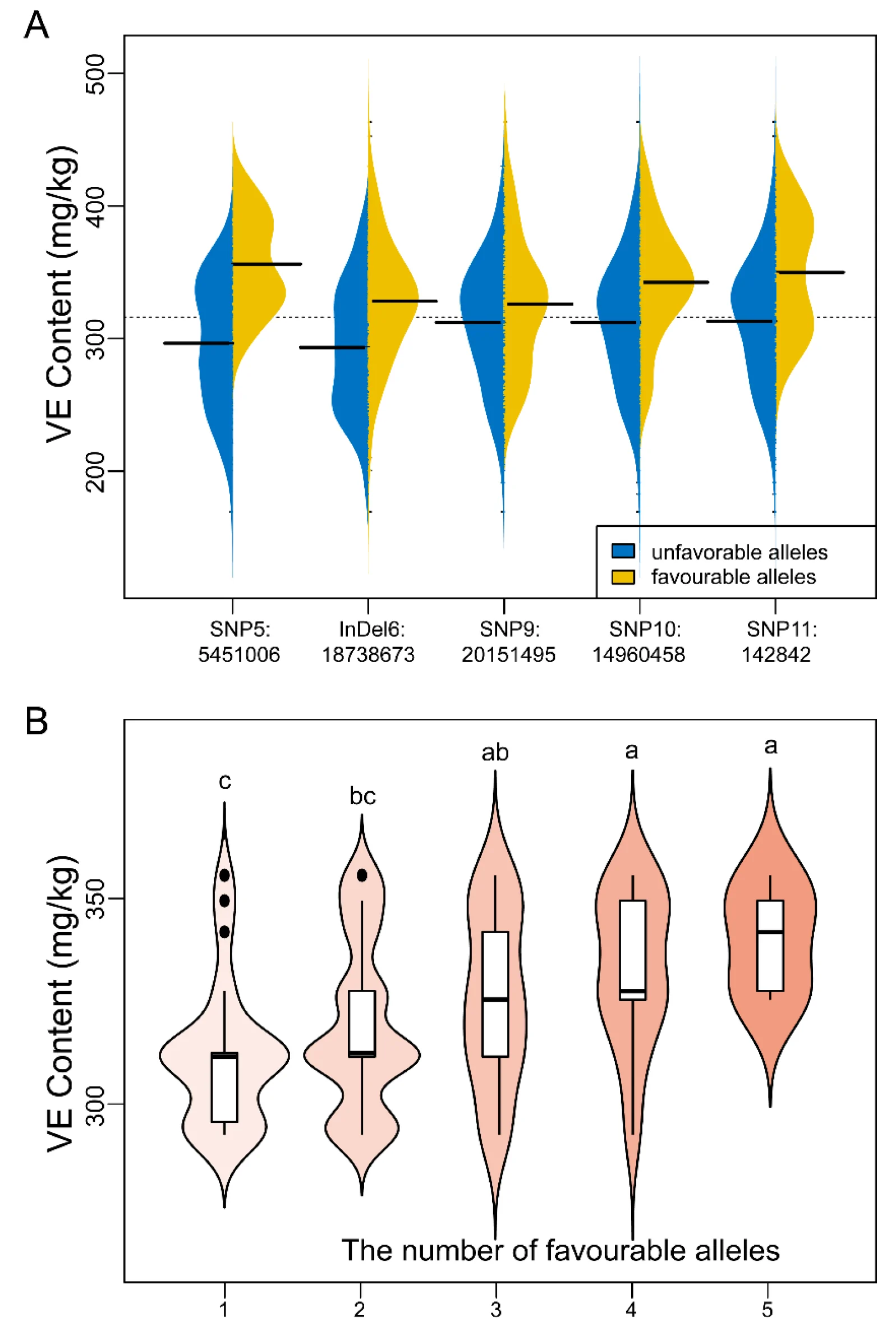
Allelic effect of significant associated loci for VE content of sesame seeds. (**A**) Beanplot showing the effect of two alleles of the significant loci. The black line represents the median value. The horizontal dashed line represents the overall mean VE content. (**B**) Effect of pyramiding favorable alleles for VE content of the significant loci. The black line in the middle of the box represents the median value, the white box represents the range from the lower to upper quartiles, and the black line represents the dispersion and frequency distribution of the phenotypic data. Black dots represent extreme sample values. Different lowercase letters (a, b, c) indicate significant differences at the *P* < 0.01 level, LSD test. The mean values of VE content in sesame under two environmental conditions were used to generate the beanplot and violin plot.

To identify core genetic loci associated with sesame VE content, 5 loci repeatedly detected across two environments or models were designated as candidates for further investigation (Table 2). Among these 5 significant associated loci, only 2 (SNP11:142842 and SNP9:20151495) were jointly identified by all three statistical models (MLM, FarmCPU, and 3VmrMLM). The phenotypic variance explained (PVE) by the significant loci ranged from 2.39% to 9.2%, with the SNP11:142842 locus exhibiting the greatest contribution. Subsequent analysis of the effects of favorable alleles at these 5 significant loci on sesame VE content revealed that all these favorable alleles contributed to an increase in VE accumulation in sesame (Figure 3A, Table S5). Furthermore, a clear trend was observed wherein the VE content of sesame accessions rose progressively as the number of accumulated favorable alleles increased (Figure 3B).

### 3.3. Comparative Transcriptome Analysis of Sesame Accessions with Differing VE Contents

**Figure 4:**
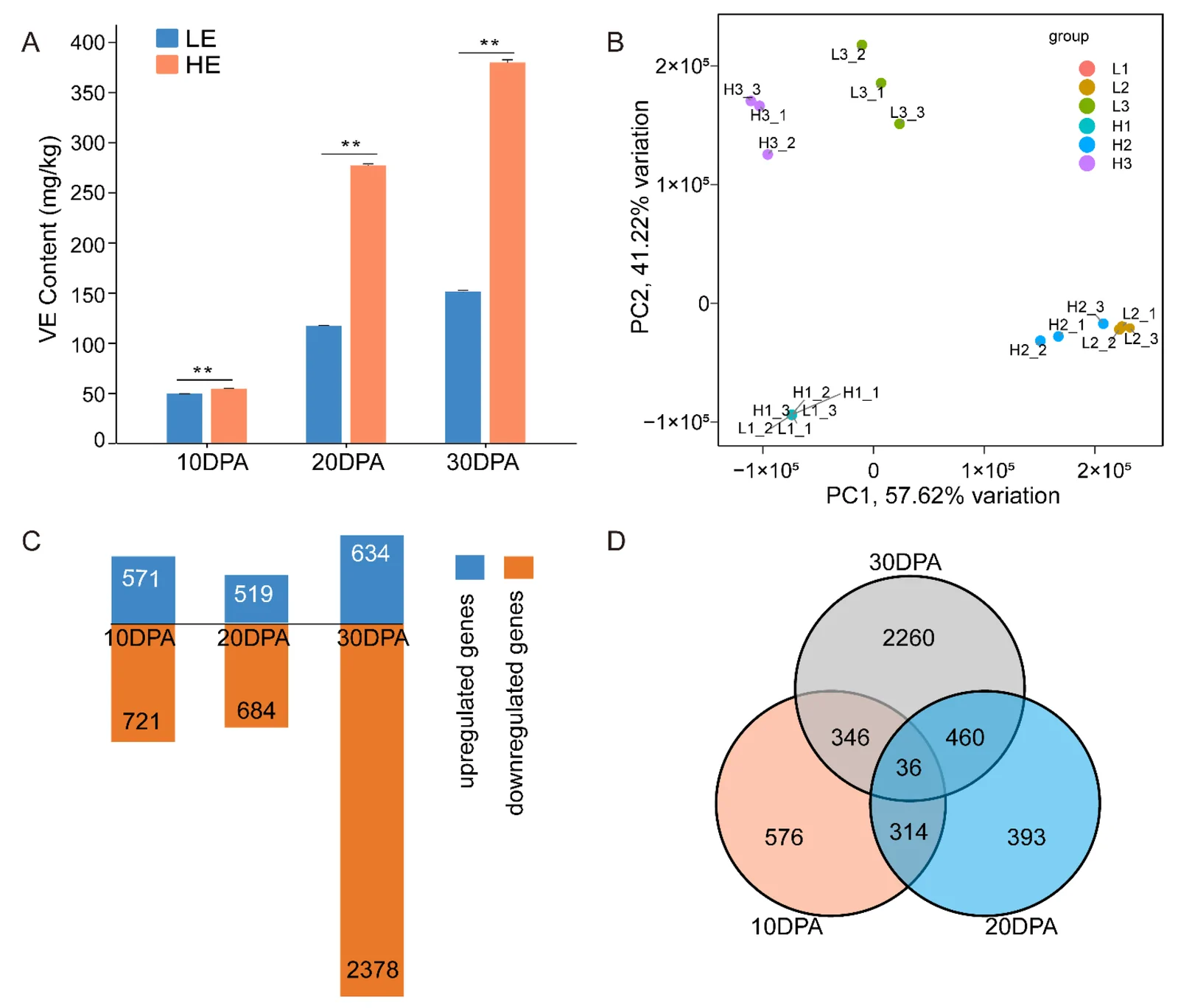
Transcriptome analysis of two sesame accessions with distinct VE contents. (**A**) The VE content in the immature seeds of low-VE-content (LE) and high-VE-content (HE) accessions at 10, 20, and 30 DPA. **, *P* < 0.01, *t*-test. (**B**) Principal component analysis (PCA) of the RNA-seq datasets. L1, L2, L3 and H1, H2, H3 represent sesame seeds from LE and HE sesame accessions at 10, 20, and 30 DPA, respectively. (**C**) Number of up- and down-regulated genes at each seed developmental stage. (**D**) Venn diagrams of differentially expressed genes. DPA, days post-anthesis.

Two sesame accessions with differing VE content, designated as high-VE-content (HE) and low-VE-content (LE) accessions, were chosen for comparative transcriptome analysis. The HE accession exhibited VE contents in mature seeds of 358.79 mg/kg (Wuhan) and 435.92 mg/kg (Linqing), while the LE accession showed contents of 151.58 mg/kg and 213.79 mg/kg in the respective environments. As illustrated in Figure 4A, the VE content in HE accession was significantly higher than that in LE accession across various developmental stages of immature seeds, validating their selection as contrasting accessions. To investigate the molecular mechanisms responsible for variations in VE content, a comparative transcriptome analysis was conducted on HE and LE varieties at three critical seed developmental stages: 10, 20, and 30 days post-anthesis (DPA). Additionally, the correlation coefficient between the three biological replicates of each sample exceeded 0.99 (Figure S2), which fully verifies the high repeatability and reliability of the experimental results.

Principal component analysis (PCA) was conducted to differentiate the transcriptomic profiles of developing seeds between the two sesame accessions at different developmental stages. The analysis identified distinct gene expression patterns between the accessions and indicated that the variations among developmental stages within the same accession were more pronounced than those between different accessions (Figure 4B), which supports the validity of sample grouping in the high-throughput sequencing experiment. Pairwise differential expression comparison between the LE and HE accessions at 10, 20, and 30 DPA yielded 1292, 1203, and 3012 DEGs, respectively (Figure 4C). DEGs varied with seed development stages, exhibiting a pattern of initial decrease followed by subsequent increase. A Venn diagram analysis identified 36 genes that were consistently differentially expressed between the two accessions throughout the entire seed development process (Figure 4D). Notably, a greater number of DEGs were identified at 30 DPA, indicating that the late stages of seed maturation are critical for VE biosynthesis.

**Figure 5:**
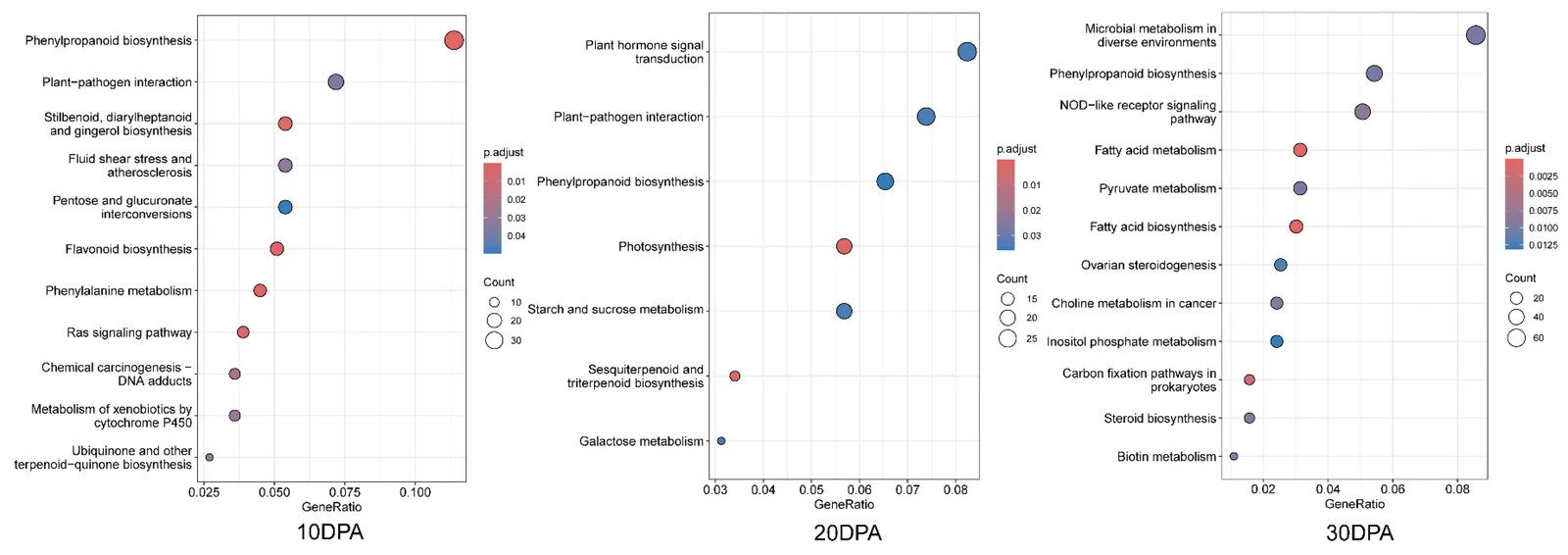
KEGG enrichment of DEGs between low-VE-content (LE) and high-VE-content (HE) accessions at 10, 20 and 30 days post-anthesis (DPA).

KEGG enrichment analysis of genes that were differentially expressed showed a notable enrichment in phenylpropanoid biosynthesis throughout all three developmental stages (Figure 5). The enrichment was most significant and involved the largest number of differentially expressed genes at 10 DPA. At 20 DPA, plant hormone signal transduction was predominantly differentially regulated, while at 30 DPA, the primary processes showing differential regulation were fatty acid metabolism and microbial metabolism in diverse environments (Figure 5). GO (gene ontology) analysis indicated that the DEGs at 10 DPA were predominantly enriched in secondary metabolic processes and the phenylpropanoid metabolic pathway. At 20 DPA, the primary enriched GO terms were related to the response to wounding. At 30 DPA, the GO terms with the highest enrichment were lipid biosynthetic process and secondary metabolic process (Figure S3). This reflects that during different developmental stages of plants, the focus of gene expression regulation varies, and the biological processes involved also differ. These findings raise the possibility that altered regulation of phenylpropanoid biosynthesis is associated with variations in sesame seed γ-tocopherol content, warranting further metabolomic validation.

To corroborate the RNA-seq findings, eight DEGs were randomly chosen, and their relative expression was measured using qRT-PCR. The outcomes aligned with the RNA-seq analysis, verifying the reliability of the transcriptomic data (Figure S4).

### 3.4. Expression of Genes Involved in the VE Biosynthetic Pathway During Seed Development in HE and LE Accessions

**Figure 6:**
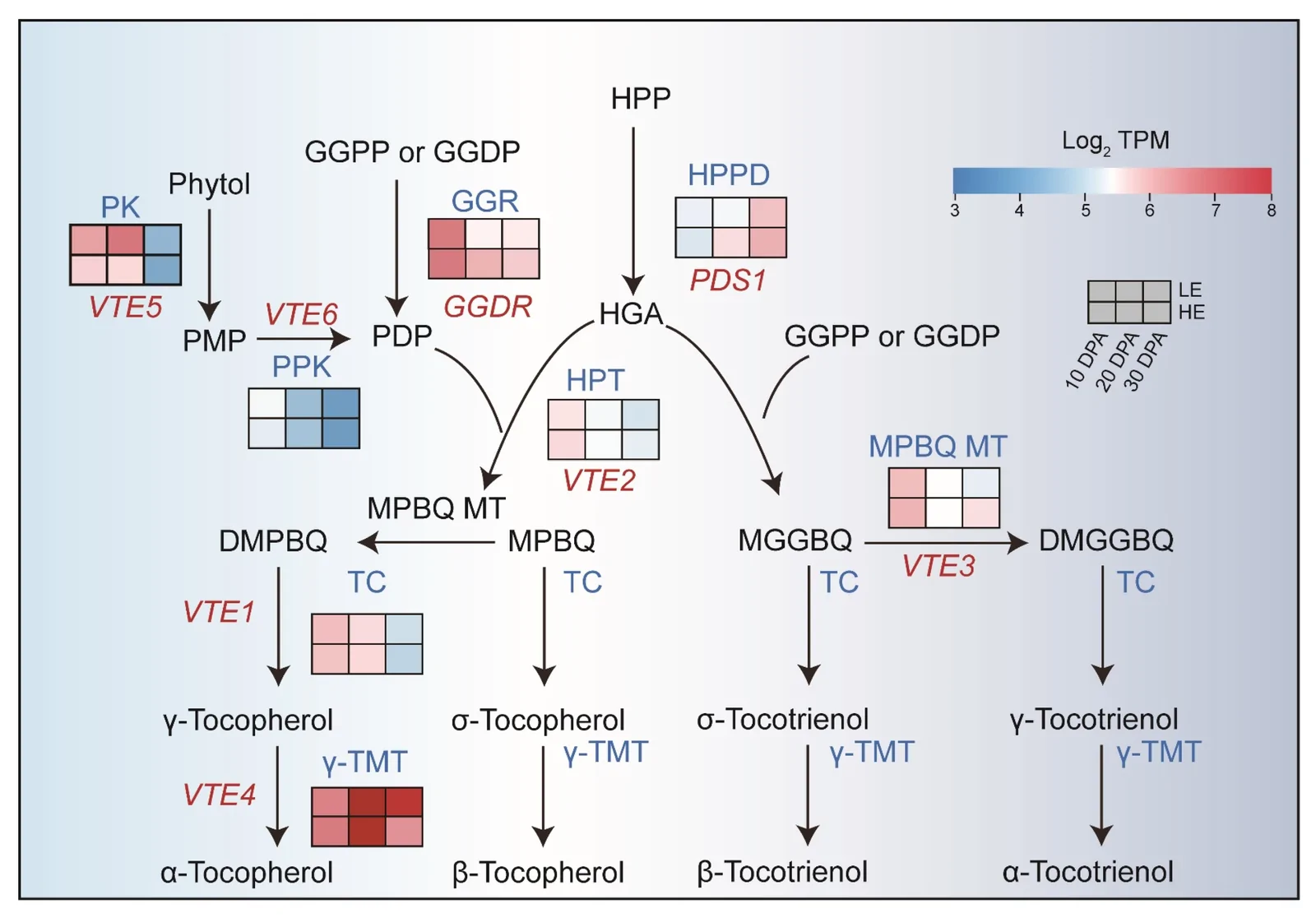
Comparison of gene expression levels in the VE biosynthesis pathway between HE and LE sesame developmental stages. Heatmap-based visualization of gene expression profiles utilizing Log_2_TPM values to sample and developmental stage comparisons. The enzyme and the gene encoding the enzyme are represented in blue and red letters, respectively. DPA, days post-anthesis. PK, Phytol kinase; PMP, Phytyl phosphate; PDP, Phytyl diphosphate; PPK, Phytyl phosphate kinase; GGPP or GGDP, geranylgeranyl pyrophosphate; GGR, geranylgeranyl reductase; HPP, p-hydroxyphenylpyruvate; HPPD, 4-p-hydroxyphenylpyruvate dioxygenase; HGA, Homogentisic acid; HPT, homogentisate phytyltransferase; MPBQ MT, 2-methyl-6-phytyl-1,4-benzoquinol methyl transferase; MPBQ, 2-methyl-6-phytylbenzoquinol; DMPBQ, 2,3-dimethyl-6-phytyl-1,4-benzoquinol; MGGBQ, 2-methyl-6-geranylgeranylbenzoquinol; DMGGBQ, 2,3-dimethyl-6-geranylgeranyl-1,4-benzoquinol; TC, Tocopherol cyclase; γ-TMT, γ-tocopherol methyl transferase.

During tocopherol biosynthesis, p-hydroxyphenylpyruvate (HPP) is transformed into different tocopherol forms by the action of multiple enzymes, including HPPD, HPT, MPBQMT, TC, and γ-TMT [42]. To dissect the molecular mechanism of differential accumulation of VE in LE and HE sesame accessions, the VE biosynthesis genes in sesame were identified through homology comparison at the whole-genome level, and their expression profiles were extracted from the RNA-seq data for comparative analysis. A total of 19 genes encoding 8 enzymes involved in the biosynthesis of VE were identified in sesame. Their detailed information and expression data are listed in Table S6. As shown in Figure 6, genes involved in VE biosynthesis showed no differential expression during early seed development (10 DPA). However, variations in expression levels of certain genes are observed during the middle and late stages of seed development. Within the VE biosynthesis pathway, HPT serves as the rate-limiting enzyme, with a core function of catalyzing the conversion of precursors of VE (HGA, PDP, and GGDP) into various tocopherol forms. These critical biosynthetic processes are catalyzed by enzymes encoded by genes such as *GGDR*, *PDS1*, and *VTE2*. Notably, the transcriptional levels of *GGDR*, *PDS1*, and *VTE2* were detected to be higher in the seeds of HE compared to those of LE at 20 and 30 DPA. The *VTE4* gene, responsible for encoding γ-TMT, which catalyzes the conversion of γ-tocopherol, was notably downregulated in HE. The differential expression of key genes related to VE biosynthesis, such as *GGDR*, *PDS1*, *VTE2*, and *VTE4*, may collectively promote the accumulation of more γ-tocopherol in HE accession.

### 3.5. Candidate Genes Identification for the VE Content in Sesame Seeds

To identify candidate genes linked to VE content, genes within a ±89 kb window around five reliably associated loci were extracted (Table S7). Subsequently, we created a Venn diagram to identify and filter overlapping genes by intersecting locus-associated genes with various DEG sets (Figure S5). Finally, based on gene annotation information, two genes (*SINPZ0901927* and *SINPZ1100015*) were selected as candidate genes involved in VE metabolism in sesame. Notably, these two genes are colocalized with the peak SNPs of the significant loci identified across all three models (SNP9:20151495 and SNP11:142842). Consequently, these two genes were identified as the primary candidates contributing to VE content variation in sesame and were selected for further analyses.

**Figure 7:**
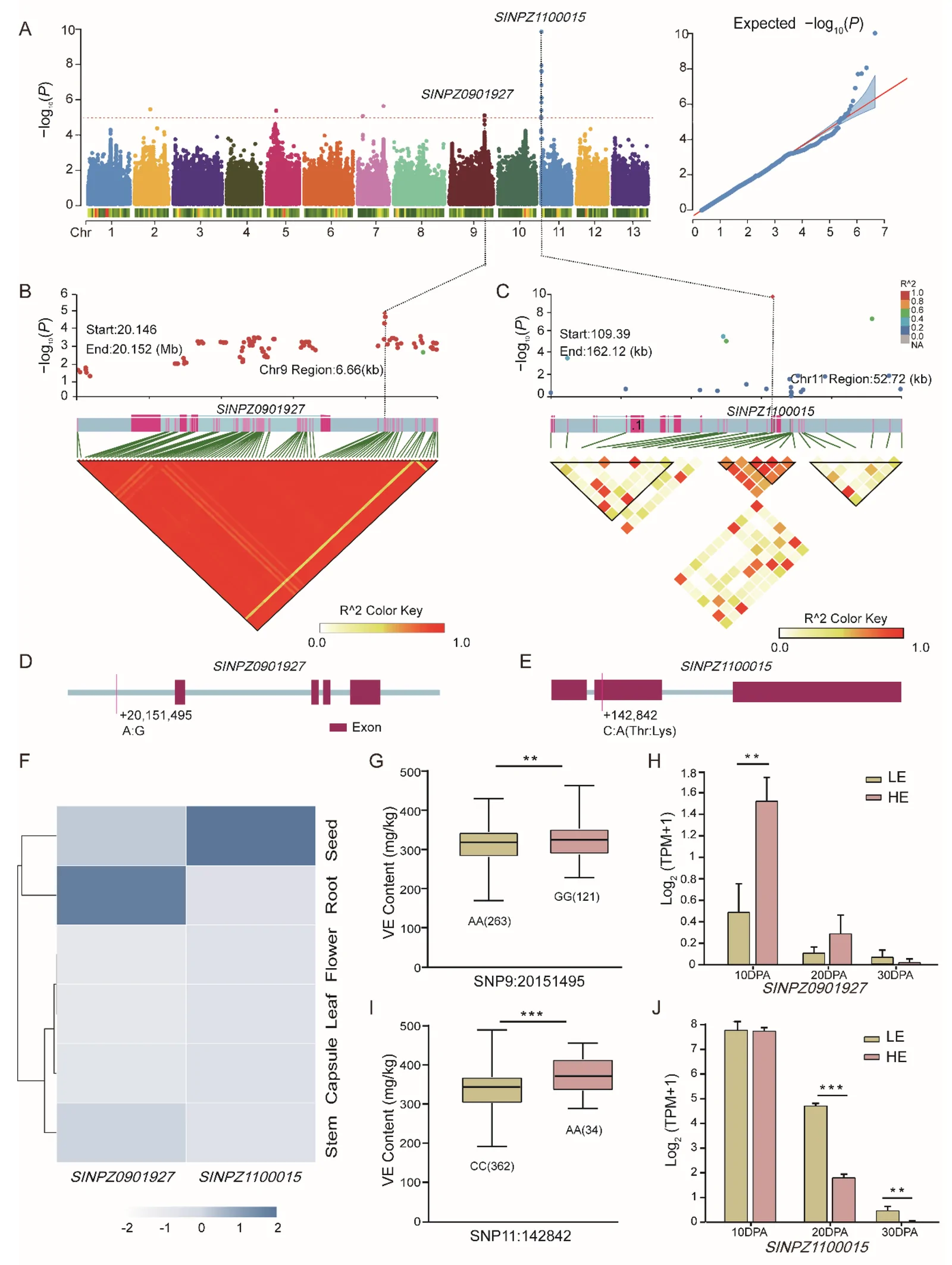
Candidate genes related to the VE content. (**A**) Manhattan plot for GWAS on VE content. The red dashed line indicates the threshold −log10(*P*) = 5; In the right-hand QQ plot, the red line represents the distribution of *P*-values under the null hypothesis; The shaded area indicates the 95% confidence interval under the null hypothesis. (**B**,**C**) LD heat map around *SINPZ0901927* (**B**) and *SINPZ1100015*/*SiNST1* (**C**). (**D**,**E**) Gene structures of *SINPZ0901927* (**D**) and *SINPZ1100015*/*SiNST1* (**E**). Red rectangle indicates exons; the red lines indicate common base mutations and non-synonymous mutations. (**F**) Heatmap of tissue expression patterns of *SINPZ0901927* and *SINPZ1100015*/*SiNST1*. (**G**,**I**) The effect of the favorable alleles in *SINPZ0901927* (**G**) and *SINPZ110015*/*SiNST1* (**I**) on VE content. The box boundary represents 0.25 and 0.75 quantiles, and the bold line represents the median value. (**H**,**J**) Expression analysis of *SINPZ0901927* (**H**) and *SINPZ1100015*/*SiNST1* (**J**) in developing seeds based on RNA-seq data. HE, high-VE-content accession. LE, low-VE-content accession. DPA, days post-anthesis. **, *P* < 0.01; ***, *P* < 0.001, *t*-test.

*SINPZ0901927*, with a length of 3680 bp, encodes a MYB transcription factor. The SNP9:20151495 was localized closest to the gene *SINPZ0901927* (Figure 7A,D), and results from linkage disequilibrium (LD) analysis provided support for this association (Figure 7B). Analysis of the peak SNPs revealed that the variant allele “G” significantly increased VE content in sesame seeds compared to the common allele “A” (*P* < 0.01; Figure 7G). Further analysis of its expression patterns across different tissues (Figure 7F) revealed that *SINPZ0901927* was predominantly expressed in roots, stems, and seeds. A significant difference in the expression of *SINPZ0901927* was observed between LE and HE accessions at 10 DPA (Figure 7H).

*SINPZ1100015* encodes a NAC transcription factor, with a length of 1438 bp and containing three exons. Its homolog in *Arabidopsis*, NST1, is a key transcriptional activator for the secondary wall biosynthesis [43]. The SNP11:142842 represented the most significant associated locus detected in both MLM and FarmCPU models and could be consistently identified across the two experimental environments (Wuhan and Linqing). The SNP11:142842 is situated in the second exon of *SINPZ1100015* (*SiNST1*) and involves a non-synonymous mutation that changes threonine (Thr) to lysine (Lys) (Figure 7A,E). Results from LD analysis provided further support for this association (Figure 7C). Analysis of the peak SNPs revealed that the variant allele “A” significantly increased the VE content in sesame seeds compared to the common allele “C” (*P* < 0.0001) (Figure 7I). Tissue expression profile analysis further revealed that *SiNST1* was specifically expressed in seeds (Figure 7F). Expression of *SiNST1* was notably elevated in LE compared to HE accessions during seed development (Figure 7J).

### 3.6. Overexpression of SiNST1 in Hairy Root of Sesame Reduced the VE Content

**Figure 8:**
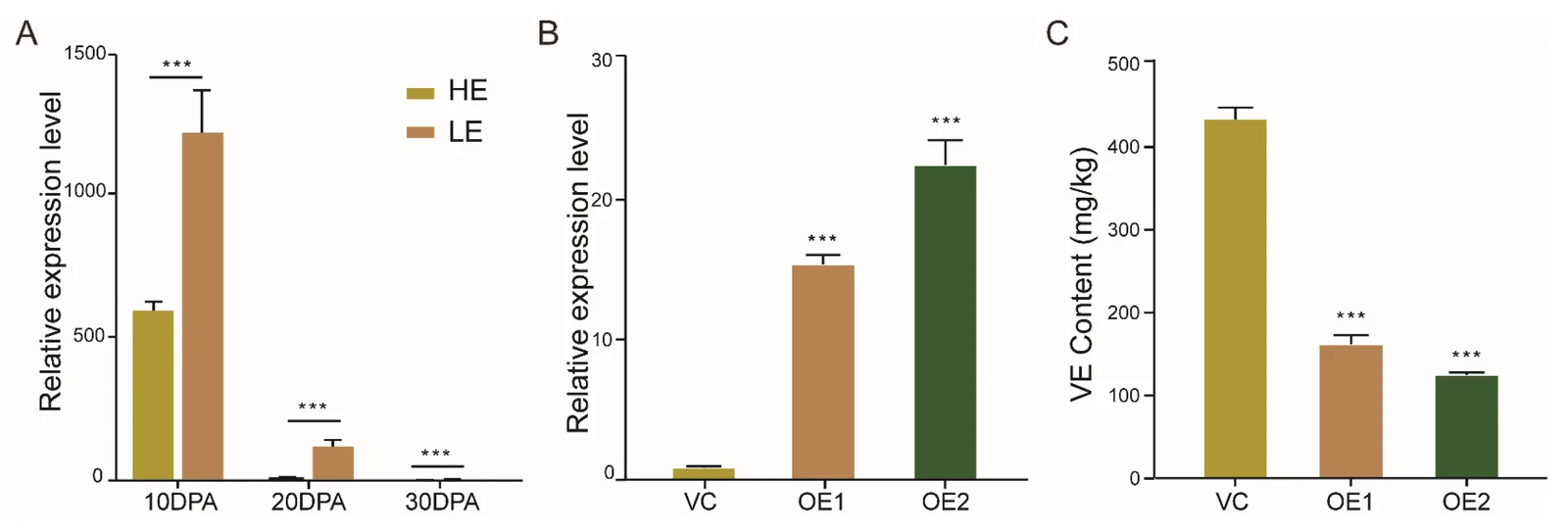
Functional verification of *SiNST1* in regulating VE content. (**A**) Expression analysis of *SiNST1* in sesame accessions with high (HE) and low VE content (LE) during seed development by qRT-PCR. DPA, days post-anthesis. (**B**) Expression analysis of *SiNST1* in transgenic hairy root and empty vector control (VC). (**C**) VE content of *SiNST1*-overexpressing sesame hairy root (*n* = 3). Data are presented as mean ± SE. ***, *P* < 0.001, *t*-test.

First, we analyzed the expression pattern of *SiNST1* at different developmental stages in seeds of LE and HE sesame via qRT-PCR. The findings indicated that *SiNST1* expression was markedly elevated during the early stages of seed development compared to the later stages. Additionally, throughout seed development, the expression level of *SiNST1* in LE was notably higher than in HE (Figure 8A).

To investigate the potential regulatory role of *SiNST1* in VE biosynthesis, *SiNST1* was overexpressed in the transgenic hairy root of sesame under the control of the 35S promoter. Two *SiNST1*-overexpressing transgenic hairy root lines (OE1 and OE2) were selected for VE content analysis by UHPLC (Figure 8B). The VE content in the *SiNST1*-OE hairy root lines was significantly lower, ranging from 124.63 to 176.66 mg/kg, compared to the VC lines (Figure 8C). Collectively, these findings indicate that *SiNST1* negatively regulates the content of VE in sesame.

## 4. Discussion

### 4.1. Characteristics of VE Content in Different Oilseed Crops

Vitamin E serves as the most efficient natural fat-soluble antioxidant and is broadly applied in industries like food preservation, medical fields, and cosmetic products. In oil crops, VE comprises eight forms: four tocopherols (α, β, γ, δ) and four tocotrienols (α, β, γ, δ). The content and proportion of different forms of VE vary in different types of crops, although γ-tocopherol dominates in most cases [44]. Rapeseed and soybean primarily contain γ-tocopherol and α-tocopherol, with γ-tocopherol comprising about 60% of total tocopherols; peanuts have similar levels of α-tocopherol and γ-tocopherol [45,46,47]; and sesame seeds are predominantly composed of γ-tocopherol, making up over 95% of their tocopherol content [14]. Common edible vegetable oils contain smaller amounts of β-tocopherol and δ-tocopherol. Tocotrienols are typically found as one or a few isomers in a mixed form in monocotyledonous plant oils like palm oil, rice bran oil, and annatto oil [48]. Among the common oilseed crop seeds, the average total VE content in soybean seeds was about 263 mg/kg [47]; the average total VE content of peanut seeds, rapeseed, and Sunflower seeds was about 135 mg/kg, 363 mg/kg and 120 mg/kg, respectively [49,50]. In the present study, only γ-tocopherol was detected in sesame seeds, with concentrations ranging from 169.33 to 463.31 mg/kg and an average of 316.28 mg/kg, positioning sesame as a superior natural source of this lipid-soluble antioxidant. Given that γ-tocopherol exhibits superior antioxidant efficacy compared to α-tocopherol [51,52], this difference may be attributed to its distinct molecular configuration and electronic properties as revealed by density functional theory [53]. The abundant accumulation of γ-tocopherol in sesame underscores its potential as a dietary antioxidant ingredient to enhance the oxidative stability of food products and support daily dietary antioxidant intake.

### 4.2. Transcriptome Analysis of HE and LE Accessions

To identify genes associated with VE metabolism in sesame seeds, we conducted a transcriptome analysis by comparing accessions with high and low VE content (HE and LE). This analysis revealed that 4474 unique genes exhibited differential expression between the two accessions during different stages of seed development. The expression of genes in the VE biosynthetic pathway was also examined during different developmental phases of HE and LE sesame seeds. VE biosynthetic pathways were notably more active during the later stages of seed development. It is worth noting that a key gene, *VTE1*, regulates the catalytic synthesis of γ- and δ-tocopherols. Numerous studies have established that VTE1 expression levels vary substantially between accessions with high and low VE contents. Overexpressing *VTE1* can enhance total tocopherol content by 4–10 times in transgenic tobacco leaves [54], *Arabidopsis thaliana* leaves [55], sweet potato [56], and *Brassica napus* seeds [57]. However, in the present study, no significant difference in *VTE1* expression levels was observed between the two sesame accessions (HE and LE); the findings are consistent with the results reported in [58]. These observations suggest that differences in the expression levels of *VTE4* and other key enzyme genes (*GGDR*, *PDS1*, *VTE2*) may be the primary cause of variations in VE content among sesame accessions. The differential expression of these genes likely modulates the overall antioxidant capacity of sesame seeds by altering the flux through the VE biosynthetic pathway. Nevertheless, the precise molecular mechanism underlying the extremely low α-tocopherol content in sesame remains unresolved and requires further investigation.

### 4.3. Transcription Factor-Mediated Regulation of Metabolic Pathway Underlying VE Content in Sesame

Integrating GWAS with transcriptomics is an effective method for pinpointing candidate genes linked to key agronomic traits. This study combined GWAS and DEGs analysis to identify two key candidate genes, both encoding transcription factors, responsible for natural variation in VE contents in sesame. Among them, *SINPZ0901927* encodes a MYB transcription factor. Beyond its established role in regulating the phenylpropanoid pathway for phenolic antioxidant synthesis such as lignin and flavonoids, the MYB family is increasingly recognized for its systemic coordination of plant antioxidant networks [59,60]. The products of the phenylpropanoid pathway and homogentisic acid (HGA) both originate from the plant aromatic amino acid metabolic network, and together, they constitute the metabolic precursor pool for VE biosynthesis [61]. Through the coordination of system-wide regulatory mechanisms, MYB factors may indirectly modulate the production of HGA and VE.

*SINPZ1100015* (*SiNST1*) encodes a NAC transcription factor. NAC proteins are a major class of plant-specific transcription factors that play a pivotal “commanding” role in regulating plant growth and development, senescence, as well as responses to both biotic and abiotic stresses [62,63]. NAC domain-containing proteins have been reported to regulate the accumulation of lignin and flavonoids by modulating the expression of rate-limiting enzyme genes (such as *PAL* and *4CL*) in the phenylpropanoid metabolic pathway, and also to control the synthesis of starch and storage proteins [29,64,65]. As a lipid-soluble antioxidant, VE plays a key role in mitigating oxidative damage, while NAC transcription factors act as master regulators of the oxidative stress response and may potentially modulate VE biosynthesis in an indirect manner [66]. These results suggest that MYB and NAC transcription factors not only govern phenylpropanoid metabolism but also modulate VE biosynthesis. This reveals a coordinated transcriptional network governing the antioxidant defense arsenal of sesame seeds, providing genetic targets to bolster the crop’s resilience against oxidative stress.

### 4.4. SiNST1 Plays a Crucial Role in Regulating Multiple Seed Traits in Sesame

*SiNST1* encodes a NAC transcription factor and is the homolog of NST1 in *Arabidopsis*. *AtNST1* has been demonstrated to regulate secondary cell wall deposition by controlling monosaccharide biosynthesis, such as cellulose and lignin, in response to multiple hormonal signals [67,68,69]. Previous studies found that *SiNST1* was also significantly associated with variations in multiple nutritional and structural traits of sesame, including oil content, fatty acid composition, seed coat thickness, protein, phytosterol, and lignans content. The variant “A” allele of SNP11:142842 in *SiNST1* is linked to a significant reduction in oil, protein, campesterol, sesamin and sesamolin levels in sesame seeds, while it correlates with increased levels of VE content, palmitic acid, linolenic acid, stigmasterol, lignin and seed coat thickness [29,40,70,71]. These findings are consistent with the previously reported trends in correlations among multiple nutritional traits in sesame [72]. Moreover, studies indicate that γ-tocopherol in sesame seeds can match the VE activity of α-tocopherol due to its synergistic interaction with sesame seed lignans [73]. Unique bioactive constituents of sesame, sesamin and sesamolin, exert this synergistic effect, effectively compensating for the low activity of γ-tocopherol in this species. These findings demonstrate that the *SiNST1* is essential in regulating the biosynthesis of multiple nutrients in sesame seed.

Collectively, the negative regulatory role of *SiNST1* in VE accumulation, coupled with its pleiotropic effects on multiple quality traits, makes it a promising target for marker-assisted breeding aimed at optimizing the nutritional and antioxidant properties of sesame products. Furthermore, the functional markers developed from the *SiNST1* locus could enable rapid screening of germplasm with superior antioxidant properties, thereby accelerating breeding efforts to enhance the health-promoting potential of sesame-based products against oxidative stress-related disorders.

### 4.5. Limitations and Future Perspectives

This study is subject to certain limitations that warrant consideration. The field experiments were conducted over a single growing season at two locations, which may not adequately represent the interannual variation in VE accumulation. Consequently, future studies should incorporate multi-year and multi-location trials to validate the stability of the identified loci. In addition, although the repressive role of *SiNST1* in VE biosynthesis was validated through hairy root overexpression assays, further investigations are necessary to determine whether directly editing *SiNST1* in sesame can effectively enhance VE content. Moreover, the additional candidate genes identified in this study require functional characterization. Such endeavors would facilitate the translation of these findings into practical breeding strategies aimed at developing sesame varieties with enhanced antioxidant capacity.

## 5. Conclusions

In summary, the study characterized the variation in VE content of 400 sesame accessions across two environments, revealing significant environmental effects on VE accumulation. Five loci significantly associated with VE content were identified, potentially aiding genetic selection and quality improvement of sesame. By integrating the results of GWAS, gene annotation and transcriptome data, two major candidate effector genes (*SINPZ1100015*/*SiNST1* and *SINPZ0901927*) were identified, with *SiNST1* being particularly noteworthy. These findings establish a crucial foundation for deciphering the molecular mechanisms governing VE biosynthetic regulation in sesame and provide valuable genetic resources for breeding varieties with enhanced VE content, which may serve as a dietary source of antioxidants for nutritional applications. Future research should prioritize multi-year, multi-environment validation of the identified loci and perform gene editing on *SiNST1* in sesame to further elevate seed γ-tocopherol levels.

## Figures and Tables

**Table 1:** Statistical analysis of VE content in sesame natural population under different environments.

Trait	Environment	Max	Min	Mean	SD	CV (%)
VE (mg/kg)	Wuhan	483.26	151.59	279.17	45.67	16.36
	Linqing	523.00	199.52	354.62	62.87	17.73

Min, minimum; Max, maximum; SD, standard deviation; CV, coefficient of variation.

**Table 2:** The significant associated loci for VE content.

Loci	Chr.	Position	Allele	*P* Value	Environment	Model/Method
SNP5: 5451006	5	5451006	G/T	5.07 × 10^−6^	Wuhan	MLM
				4.42 × 10^−9^	Wuhan	FarmCPU
InDel6: 18738673	6	18738673	CAA/C	1.54 × 10^−6^	Lingqing	FarmCPU
				5.49 × 10^−30^		3VmrMLM
SNP9: 20151495	9	20151495	A/G	7.14 × 10^−6^	Wuhan	MLM
				1.63 × 10^−11^	Wuhan	FarmCPU
				2.99 × 10^−30^		3VmrMLM
SNP10: 14960458	10	14960458	T/C	1.35 × 10^−6^	Lingqing	MLM
				2.84 × 10^−7^	Lingqing	FarmCPU
SNP11: 142842	11	142842	C/A	1.71 × 10^−10^	Wuhan	MLM
				8.39 × 10^−17^	Wuhan	FarmCPU
				6.31 × 10^−6^	Lingqing	FarmCPU
				7.78 × 10^−33^		3VmrMLM

## Data Availability

The RNA-seq reads data reported in this paper have been deposited in the Genome Sequence Archive in National Genomics Data Center, China National Center for Bioinformation/Beijing Institute of Genomics, Chinese Academy of Sciences (GSA: CRA046961) that are publicly accessible at https://ngdc.cncb.ac.cn/gsa (accessed on 24 July 2026).

## Supplementary Materials

The following supporting information can be downloaded at https://www.mdpi.com/article/10.3390/antiox15091088/s1, Figure S1: QQ plot of GWAS results in the Linqing (LQ) and Wuhan (WH) environments; Figure S2: The correlation coefficient between experimental samples, three replicates per sample; Figure S3: GO enrichment of DEGs between low-VE-content (LE) and high-VE-content (HE) sesame accessions at 10, 20 and 30 days post-anthesis (DPA); Figure S4: Validation of transcriptome sequencing results by qRT-PCR; Figure S5: The overlap of candidate genes from GWAS and transcriptome analysis results. Table S1: The list of the 400 sesame accessions used in this study; Table S2: Statistical analyses and mapping results of RNA sequencing reads; Table S3: List of primers used in this study; Table S4: Vitamin E content of 400 sesame accessions grown in Linqing and Wuhan environments; Table S5: Allele effects of the significant associated loci for vitamin E content; Table S6: Expression of genes involved in vitamin E biosynthesis pathway in sesame accessions with different vitamin E contents during seed development; Table S7: List of lead loci significantly associated with VE content and the related candidate genes.
