# Supplementary material for: Genome-Wide Association Study Reveals Novel Loci and Candidate Genes of Vitamin E Content in Sesame (Sesamum indicum L.)
Source: Antioxidants (Basel). 2026 Aug 29;15(9):1088. doi: 10.3390/antiox15091088 (PMC13603810; doi:10.3390/antiox15091088)
Supplement: Supplementary file 1 [file antioxidants-15-01088-s001.zip › Supplementary Figure.pdf]

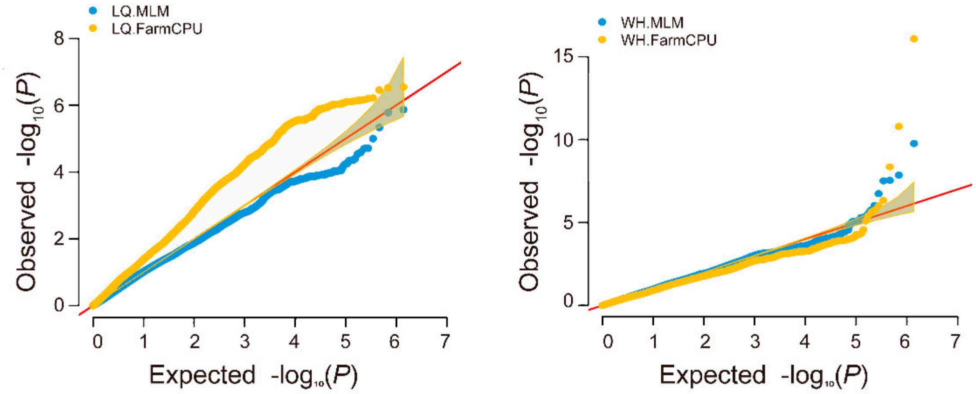

Figure S1. QQ plot of GWAS results in the Linqing (LQ) and Wuhan (WH) environments.

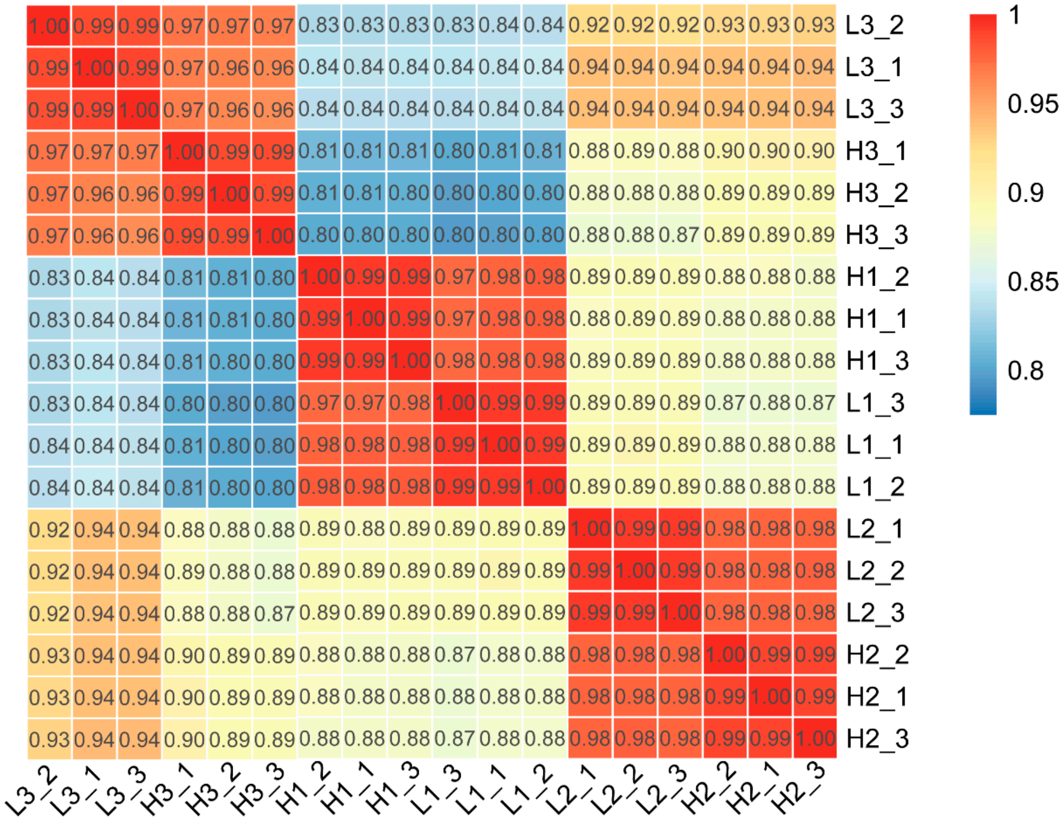

Figure S2. The correlation coefficient between experimental samples, three replicates per sample. L1, L2, L3 and H1, H2, H3 represent sesame seeds from low-VE-content (LE) and high-VE-content (HE) sesame accessions at 10 days, 20 days, and 30 days post-anthesis, respectively.

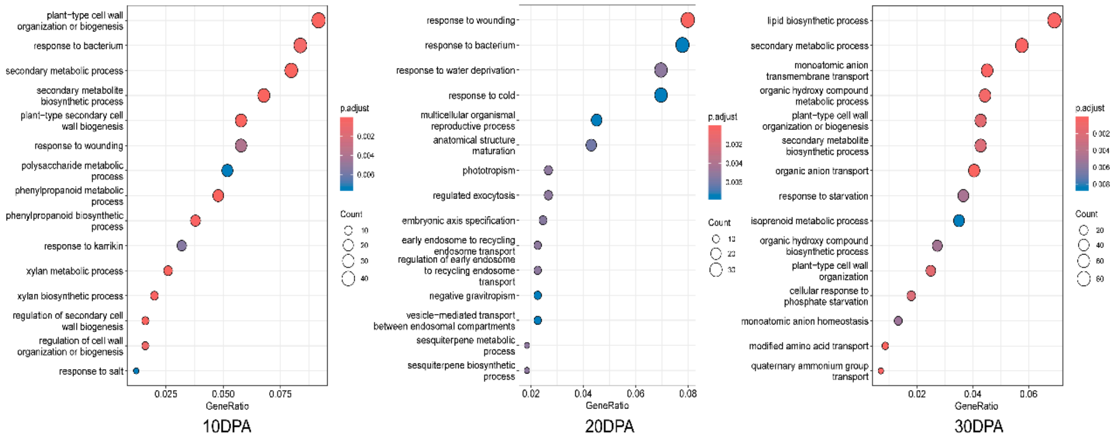

Figure S3. GO enrichment of DEGs between low-VE-content (LE) and high-VE-content (HE) sesame accessions at 10, 20 and 30 days post-anthesis (DPA).

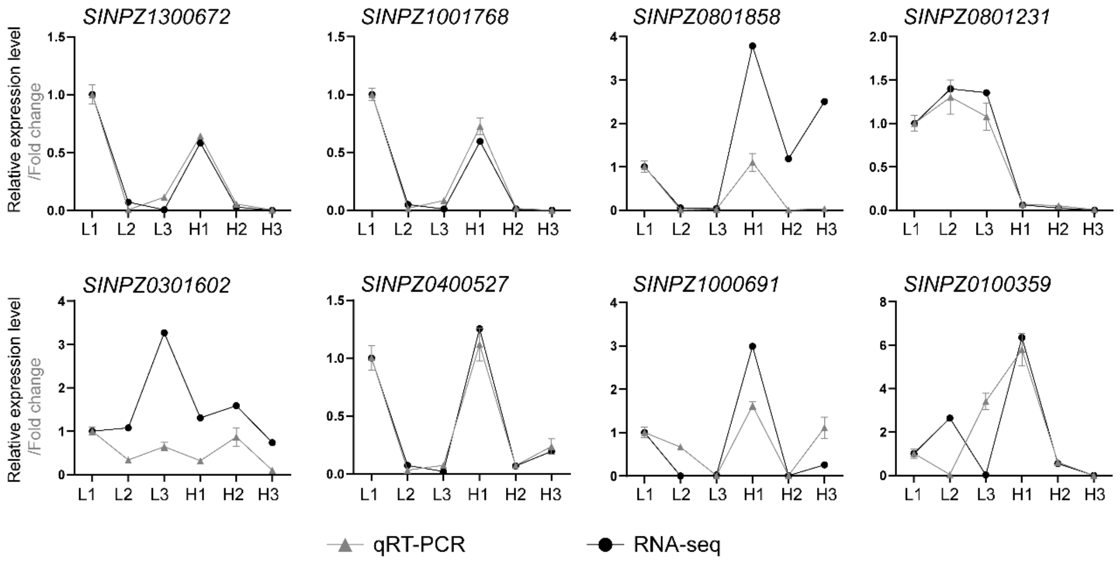

Figure S4. Validation of transcriptome sequencing results by qRT-PCR. Black circles and grey triangles represent RNA-seq and qRT-PCR results, respectively. L1, L2, L3 and H1, H2, H3 represent sesame seeds from low-VE-content (LE) and high-VE-content (HE) sesame accessions at 10 days, 20 days, and 30 days post-anthesis, respectively.

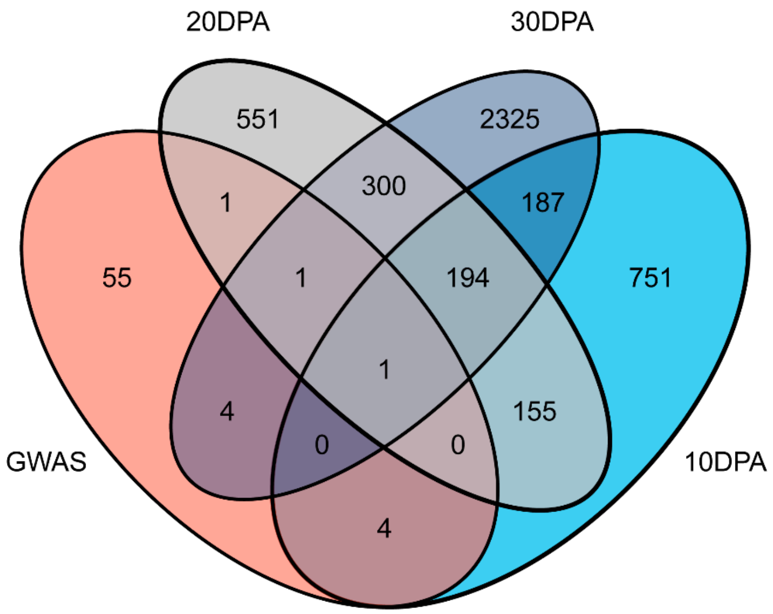

Figure S5. The overlap of candidate genes from GWAS and transcriptome analysis results.
